# The Novel C5aR Antagonist DF3016A Protects Neurons Against Ischemic Neuroinflammatory Injury

**DOI:** 10.1007/s12640-019-00026-w

**Published:** 2019-04-05

**Authors:** Laura Brandolini, Marta Grannonico, Gianluca Bianchini, Alessia Colanardi, Pierluigi Sebastiani, Antonella Paladini, Alba Piroli, Marcello Allegretti, Giustino Varrassi, Silvia Di Loreto

**Affiliations:** 1 Dompé Farmaceutici SpA, Via Campo di Pile, L’Aquila, Italy; 2 Department of MESVA, University of L’Aquila, L’Aquila, Italy; 3 Institute of Translational Pharmacology (IFT) – National Council of Research (CNR), L’Aquila, Italy; 4 Paolo Procacci Foundation, Via Tacito 7, 00193 Rome, Italy

**Keywords:** Neuroinflammation, Complement, C5a, Cortical neurons, Cytokines, Pain

## Abstract

The central nervous system (CNS) constitutively expresses complement (C) membrane receptors and complement proteins, including the component C5a. This is a crucial terminal effector of the C cascade, mostly involved in pain and neuroinflammatory conditions. Aberrant activation of C5a protein and its receptor C5aR has been reported to play a critical role in neurodegenerative diseases, with important clinical consequences. Here we have investigated the effects of DF3016A, a novel selective C5aR antagonist, able to penetrate the blood-brain barrier (BBB), on cortical neurons exposed to oxygen-glucose deprivation-reoxygenation (OGD/R), a neuroinflammation-related process. We demonstrated that a mild ischemic insult induces an early upregulation of C5aR associated with the over-production of pro-inflammatory cytokines and the over-expression of the transcriptional regulatory factor miR-181. Furthermore, we report the first experimental evidence of the effect of DF3016A, modulating complement component C5a, on neurons in a model of injury. Interestingly, DF3016A protects neuronal viability by restoring intracellular calcium levels, thus opposing the increase in pro-inflammatory cytokine levels and miR-181 expression. Based on our results, we suggest that DF3016A is a novel C5aR antagonist promoting protective effects against OGD/R-induced damage that could be a new therapeutic approach to controlling CNS neuroinflammatory conditions.

## Introduction

Neuroinflammation and neurodegenerative processes have been strongly related to chronic pain (Alexander et al. 2008; Varrassi et al. 2015). They may be consequent to the activation of complement in the central nervous system (CNS) (Van Beek et al. 2003). More recently, the importance of neurodegenerative processes has been related to concomitant pathological conditions connected to pain such as paraneoplastic neuropathies, rheumatic, and joint diseases (Fusco et al. 2017; Zis et al. 2017; Zis et al. 2017a). Despite being immunologically separate from the circulating plasma, and in the absence of blood-brain barrier (BBB) damage, cells of the CNS express many components of immunity including all complement (C) factors (Woodruff et al. 2010; O'Barr et al. 2001). The C system, composed of a large number of serum proteins and membrane-bound receptors, among others, protects from infection through innate and adaptive immune mechanisms (Carroll 1998; Song et al. 2000). In the CNS, complement component 5a (C5a) and its cell membrane receptor (C5aR) are constitutively expressed not only in astrocytes and microglia (Müller-Ladner et al. 1996) but also in neurons (Davoust et al. 1999; O'Barr et al. 2001; Pavlovski et al. 2012). The C cascade pathway is involved in pain mechanisms (Gasque et al. 1997), and the inappropriate activation of complement produces a local inflammatory reaction. Much evidence indicates that C5a and C5aR play a critical role in neurodegenerative diseases (Yanamadala and Friedlander 2010; Zhou et al. 2009; Quadros and Cunha 2016; Barnum 2002; Farkas et al. 2003), and the C5a has a number of effects on neurons in vitro (Mukherjee and Pasinetti 2001; Farkas et al. 1998; Pavlovski et al. 2012). For more than 30 years, researchers have actively investigated the role of the immune system (IS) and specifically, C proteins, in human neurodegenerative diseases and mouse models (Alexander et al. 2008). C production in the CNS not only protects from invading pathogens but also may have a regulatory role in neuronal processes (Farkas et al. 2012; Farkas et al. 2008). However, there is compelling evidence that it can exert a dual role in the CNS, depending on the pathophysiological context (van Beek et al. 2003). Stevens et al. (2007) reported a C involvement during brain development in physiological homeostasis and pruning of unwanted synapses, suggesting that the aberrant reactivation of complement-mediated synapse elimination may take place in neurodegenerative conditions (Mukherjee and Pasinetti 2000). After ischemic stroke, the progression of brain injury also involves complement mediated changes (Alawieh et al. 2015).

Acute ischemic stroke is responsible for almost 90% of neuronal injuries following deprivation of oxygen and nutrients. During the ischemic cerebrovascular events, there are two zones: the “ischemic core” and the “ischemic penumbra.” Focal ischemic stroke induces neuronal death in the “core” and molecular and cellular alterations in the “penumbra,” changes that are temporal/spatial-dependent. The term ischemic penumbra is generally used to define the tissue surrounding the severe ischemic core where blood flow is reduced to cause hypoxia, but not sufficient to result in irreversible failure of energy metabolism and cellular necrosis (Astrup et al. 1981). Ischemic penumbra is a diagnostic and biochemical target for brain plasticity, neuroprotection, and neurorepair. Moreover, a sublethal preconditioning injury strongly increases the neuronal resistance to subsequent injury by stimulating protective processes (Badaut et al. 2005; Kawahara et al. 2004; Narayanan and Perez-Pinzon 2017).

Oxygen-glucose deprivation/reoxygenation (OGD/R) procedure provides an in vitro model of neuroinflammation-related processes (Goldberg and Choi 1993) that are crucial elements in the injury onset and progression. To better understand the relevance of C5a modulation in this context, we ran a medicinal chemistry program (Moriconi et al. 2014) that led to the identification of DF3016A, a novel potent and selective C5aR inhibitor with tailored pharmacological properties. Herein, we report and evaluate the in vitro characterization and the experimental effects of DF3016A, on primary rat cortical neurons exposed to sublethal OGD/R.

## Materials and Methods

### Cell Isolation and Culture

Polymorphonuclear cells (PMNs) were obtained from buffy coats of heparinized peripheral blood from adult healthy volunteers, as previously described (Bertini et al. 2004). Ethical clearance was obtained by local ethical review committees and conformed to Italian regulations. PMNs were separated by dextran sedimentation followed by hypotonic lysis of contaminating red blood cells as previously described (Bertini et al. 2004). Cell viability, as measured by trypan blue dye exclusion, was greater than 98%.

Murine and rat PMNs were isolated from peritoneal cavities injected with 1.5 ml of 3% thioglycolate in saline to male Balb/c mice (20–25 g and 7–9 weeks of age, provided by Charles River, Calco, Italy) or with 10 ml of 1.5 ml of 3% thioglycolate in saline to SD rat (370–450 g and 3–4 months of age provided by Charles River, UK). Four hours after injection, the animals were sacrificed by decapitation and peritoneal cavities were washed with saline. PMNs were recovered and centrifuged at 600×*g* for 10 min. The pellet was resuspended in Hank’s Balanced Salt Solution (HBSS); then the cells were counted using Türk’s solution and diluted at 3 × 10^6^ cells/ml.

### Migration Assay

Migration of human, rat, and mouse PMNs was evaluated using a 48-well micro-chemotaxis chamber, as previously described (Bertini et al. 2004). Briefly, 25 μl of control medium HBSS for human and rodent PMNs, or chemoattractant solution 10 nM chemokine (CXC motif) ligand 1 (CXCL1), 1 nM CXCL8 or CXCL1/KC for rodent PMNs, 10 nM C5a or 10 nM mC5a or rC5a for rodent PMNs, 10 nM CXCL12, and 10 nM n-formylmethionyl-leucyl-phenylalanine (fMLP) were seeded in the lower compartment of the chemotaxis chamber. Fifty microliters of cell suspension (1.5 × 10^6^/ml for human PMNs, 3.0 × 10^6^/ml for rat and mouse PMNs) pre-incubated at 37 °C for 15 min in the presence or absence of different concentrations of DF3016A or vehicle was seeded in the upper compartment. The chamber was incubated at 37 °C in air with 5% CO_2_ for 45 min. At the end of incubation, filters containing migrated cells were removed, fixed, and stained with Diff-Quik, and five oil immersion fields at high magnification (×100; Zeiss microscope) were counted after sample coding.

### Peritoneal Murine Macrophage Preparation and Lipopolysaccharide-Induced Prostaglandin E2 Production

Peritoneal exudate cells were collected from peritoneal washings of male mice Balb/c mice (20–25 g and 7–9 weeks of age, provided by Charles River, Calco, Italy), 5 days after i.p. injection of 3% thioglycollate in saline (1.5 ml per mouse), as previously reported (Mascagni et al. 2000). Cells were placed at 1 × 10^6^ ml^−1^ in 96-well plates and non-adherent cells removed by gentle washing 2 h later. DF3016A was then added to adherent macrophages 20 min before adding lipopolysaccharide (LPS) (1 μg ml^−1^). Control cells received vehicle at the appropriate dilution. Total prostaglandin E2 (PGE_2_) production was determined in the supernatant 24 h after LPS stimulation. PGE_2_ levels were measured by Enzyme Immunoassay (EIA) Kit (sensitivity 2.5 pg per well).

### In Vitro Selectivity

DF3016A was tested in the SafetyScreen44 Panel performed at Eurofins Cerep SA (France) by radio ligand binding assays to assess the off-target activities towards a panel of GPCRs, enzymes, ion channels, transporters, and nuclear receptors. All selected targets are recommended by four major pharmaceutical companies (Bowes et al. 2012). The compound was tested at a single concentration of 10 μM in triplicate.

DF3016A was dissolved in dimethyl sulfoxide (DMSO) to achieve 10 mM stock solution that was diluted with water/HBSS to a final concentration of 10 μM. Cell membrane homogenates (48 μg protein) were incubated for 60 min at 22 °C with the respective reference compound in the absence or presence of the test compound in a buffer containing 50 mM Tris-HCl (pH 7.4), 2 mM MgCl_2_, and 1 mM ethylene-diamine-tetra-acetic acid (EDTA). After incubation, the samples were filtered rapidly under vacuum through glass fiber filters (GF/B, Packard Instruments, Meriden, CT, USA) presoaked with 0.3% polyethyleneimine (PEI), and rinsed several times with ice-cold 50 mM Tris-HCl using a 96-sample cell harvester (Unifilter, Packard Instruments). The filters were dried, then counted for radioactivity in a scintillation counter (Topcount, Packard Instruments) using a scintillation cocktail (Microscint-O, Packard Instruments).

### Brain Penetration in the Rat

DF3016A was studied in male Sprague Dawley rats (*n* = 3) to investigate the BBB penetration after oral administration. SD rats (370–450 g and 3–4 months of age at the time of testing) were purchased from Charles River, UK. All animals were housed in groups of three per room under controlled conditions of temperature (19 to 21 °C) and humidity (50%), and maintained on a 12-h light/dark cycle (lights on at 7 and lights off at 19 including a 30-min dawn/dusk lights increasing/dimming period, respectively). Animals had free access to rat chow pellets CRM (P) and water except during the experimental procedures. All animals were weighed immediately before the test. The test drug was administered orally at 30 mg/kg using saline solution (0.9% NaCl) as vehicle. The animals were sacrificed at *T*_max_ (2 h postdose) with sodium pentobarbital administered intraperitoneally. Cardiac blood samples, 0.6 ml, were obtained and placed in EDTA-coated tubes. The tubes were spun at 13,000 rpm for 4 min and 100 μl of supernatant taken and immediately stored at − 80 °C prior to analysis. Plasma samples were analyzed by liquid chromatography–mass spectrometry (LC-MS/MS) following extraction by protein precipitation with internal standard in acetonitrile, and levels of parent measured against an extracted calibration curve of plasma samples spiked with test compound. The whole brain was harvested then placed in a plastic tube and immediately frozen at − 80 °C until required. Brain tissue were homogenized and analyzed by LC-MS/MS following extraction by protein precipitation, and levels of parent measured against an extracted calibration curve of brain homogenate samples spiked with the test compound. Total exposure levels of the test compound in brain and plasma at 2 h postdose were determined.

### Primary Cortical Cultures

Neuronal cultures were prepared from cortices of 17–18-day-old Sprague-Dawley rat fetuses. Two pregnant rats, provided from Envigo RMS S.r.l. (Z.I. Azzida, 57–33,049 S. Pietro al Natisone, Udine Italy), were euthanized by exposition to a gradually rising concentration of CO_2_ and then decapitated. Rat brains were isolated using standard procedures, and after the elimination of meninges, the cortical tissues were dissociated by trituration and digestion with 20 U/ml papain (Invitrogen, Karlsruhe, Germany) for 30 min at 37 °C. Approximately 1 × 10^6^ cells in 1 ml DMEM/F12 medium containing 0.2 mM Glutamax, 1% penicillin/streptomycin (Pen/Strep), 5% fetal bovine serum, and 5% horse serum were seeded into poly-D-lysine pretreated plates. After 4–6 h, the medium was changed with Neurobasal containing the serum-free B27 supplement (2%), 1% Pen/Strep, and 0.2 mM Glutamax that selectively inhibits glial proliferation. Neurons were maintained in a humidified atmosphere (5% CO_2_/95% air) at 37 °C and used for experiments after 8–10 days in vitro. All experimental procedures were performed according to Italian law 116/92, authorization no. 104–2013-A.

### Drug Treatment

DF3016A was dissolved in DMSO. The final concentration of DMSO in the medium was below 0.1%. To characterize toxicity, primary cultures were treated for 24 h with different DF3016A concentrations (10 nM, 50 nM, 100 nM, 500 nM, 1 μM, 5 μM, 10 μM, 100 μM, 0.5 mM, 1 mM), finally treated with suitable concentration, and exposed to OGD/R (1 h first, and then 3 h). Control conditions underwent neither drug treatment nor OGD/R. After treatments, cultures were used for in vitro experiments. Different tests were carried out as described below.

### Experimental Model

We performed an experimental model in vitro reproducing neuroinflammation stress condition. Less than 50% mortality was induced to mimic the damage occurred in the area surrounding the ischemic core where neurons still have capacity for recovery. Previously time–response experiments conducted for more than 1 h (2 h) of OGD induced more than 50% mortality (data not shown). On this basis, we selected only 1-h OGD that induces about 20% mortality in primary neuronal cultures and allows resilience of alive neurons.

### OGD/R

DF3016A (500 nM) was added to the cortical neurons which were exposed to O_2_ and glucose deprivation with glucose-free Neurobasal in Hypoxia Chamber for Cell Culture (BioSpherix) at 5% CO_2_ atmosphere-85% N_2_. O_2_ concentration was monitored by using a ProOx sensor controller, and the 0.03% O_2_ value was considered acceptable. Cells were incubated in this anaerobic chamber for 1 h and then removed from the anaerobic environment. Hence, neurobasal media were substituted and cultures placed in 95% air and 5% CO_2_ conditions, for 3 h of reoxygenation. Cells of control group were treated identically except that they were not exposed to OGD/R.

### Cell Viability Evaluation

Quantifications of neuronal viability were assessed by MTS method using Cell Titer One Solution Cell Proliferation Assay (Promega Corporation Madison, WI, USA) based on 3-(4,5-dimethylthiazol-2-yl)-5-(3-carboxymethoxyphenil)-2-(4-sulfophenyl)-2H-tetrazolium (MTS) (dilution 1:10). Viable cells with active metabolism convert MTT into formazan, detected by absorbance measurements at 490 nm in a BioTeek Elx800 microplate reader. Acridine Orange (AO) and Propidium Iodide (PI) double staining have been used to evaluate viable and dead cells in controls and exposed neurons. Images have been acquired through a fluorescent microscope combined with a Digital Color Camera.

### Isolation of RNA and qRT-PCR

The total RNA, including microRNA (miRNA), from cells in cultures were extracted with Ribospin Kit (GenaAll) followed by Riboclear Plus Kit (GenaAll) to remove residual DNA according to the manufacturer’s protocols.

Total mRNA transcription (2μg/20 μl) was performed using High Capacity cDNA Reverse Transcription Kit (Applied Biosystems) and reverse transcription for MicroRNA (10 ng/15 μl) was performed using TaqMan MicroRNA Reverse Transcription Kit (Applied Biosystems).

The qPCR 20 μl/reactions for miR-181a (assay ID: hsa-miR-181a) and C5aR (assay ID: Rn02134203_s1) were conducted in triplicate using TaqMan® MicroRNA Assay Kit and TaqMan® Gene Expression Assays protocol (Applied Biosystems), respectively, following the manufacturer’s instructions. Reactions were performed using a Step One Plus Applied Biosystems thermocycler (Life Technologies) with the default protocol. Briefly, for each reaction 40 cycles of amplification with the following profile were performed: 95 °C for 10 min for the first cycle, followed by 40 cycles of 95 °C for 15 s, and 60 °C for 1 min.

Relative intensity of PCR-specific amplicons was calculated using the 2^−ΔΔCt^ method (Livak and Schmittgen 2001). Beta-actin (assay ID: Rn00667869_m1) and U6 (assay ID: 001973) small nuclear RNA (snRNA) (Han et al. 2015) were used as internal control to normalize the level of each transcript.

### Fluo-4 NW Calcium Assay

Neuronal cells, grown on 96-well plates, were exposed to OGD/R and treated with 500 nM of DF3016A; growth media were removed, and 100 μl of the dye loading solution (F36206 Fluo-4 NW Calcium Assay Kit, Invitrogen) was added carefully to each well. Plates were incubated at 37 °C for 30 min and then at room temperature for an additional 30 min. Fluorescence was revealed at 516 nm with Victor 3 Model 1420-012 Multi-label Microplate Reader. Data were presented as the ratio between Ca^++^ influx and neuronal viability.

### ELISAs

Quantitative measurements of cellular proteins (C5a, TNF-α, IL-1β, and IL-6) in cell culture supernatants were determined by ELISA methods using Rat IL-6, Mouse TNFα, Rat IL-1β PicoKine^™^ ELISA Kits (Boster Biological Technology) and Rat C5a ELISA Kit (Elabscience Biotechnology Inc.) according to the manufacturer’s instructions. All tests were performed in triplicate.

### Data Analysis

Data are expressed as mean ± standard error of mean (SE). Mean group differences were evaluated by one-way analysis of variance (ANOVA) followed by Bonferroni’s post hoc test. *P* values less than 0.05 were considered statistically significant. All statistical analyses were performed using SPSS 19.0 software.

## Results

### In Vitro Characterization

In vitro pharmacological characterization showed that DF3016A did not inhibit spontaneous cell migration per se yet potently inhibited the C5a-induced human PMN migration with an IC_50_ of 50 nM and cross-reactions with rat and mouse C5aR (IC_50_ = 45 nM and 37 nM, respectively). The compound exhibited a high degree of selectivity with no effect on PMN migration induced by other leukocyte activators such as CXCL8, CXCL1, CCL3, fMLP, CXCL12, and on LPS-induced PGE2 accumulation up to 10 μM concentration.

### In Vitro Selectivity

DF3016A was tested in order to assess the off-target activities towards a panel of GPCRs, enzymes, ion channels, transporters, and nuclear receptors (44 targets in total, according to the SafetyScreen44 assay provided by Eurofins Cerep). Radioligand binding assays were carried out at the test concentration of 10 μM in triplicate. We found that DF3016A did not show any inhibition of the assayed receptors, confirming the high selectivity towards the C5aR.

### Brain Penetration in Rats

With the aim of assessing the therapeutic potential of DF3016A in CNS disorders, brain penetration of the molecule was tested in SD rats after oral administration at 30 mg/kg. At 2 h postdose (*T*_max_), we found that the levels of DF3016A were 104,683 ng/ml in plasma and 4172 ng/g in brain, with a brain to plasma ratio of 0.04. Once adjusted for the free plasma and brain fractions (0.7% and 7.7%, respectively), this increases to an unbound brain to plasma ratio (Kpuu) of 0.44 (Table 1). This means that oral DF3016A is able to sufficiently penetrate the brain.

**Table 1:** Concentrations of DF3016A in plasma and brain of rats following oral dosing (30 mg/kg)

DF3016A					
rPPB Fu	rBTB Fu	PK (30 mg/kg, PO)			
		Plasma *C*_max_ (ng/mL)	Brain *C*_max_ (ng/g)	Kp	Kpuu
0.7%	7.7%	104,683 ± 8867	4172 ± 323	0.04	0.44

The values of plasma and brain concentrations are the mean ± S.D. (*n* = 3)

rPPB rat plasma protein binding, rBTB rat brain tissue binding, Kp brain to plasma ratio, Kpuu unbound brain to plasma ratio

### DF3016A Evaluation on Neuronal Viability

**Fig. 1:**
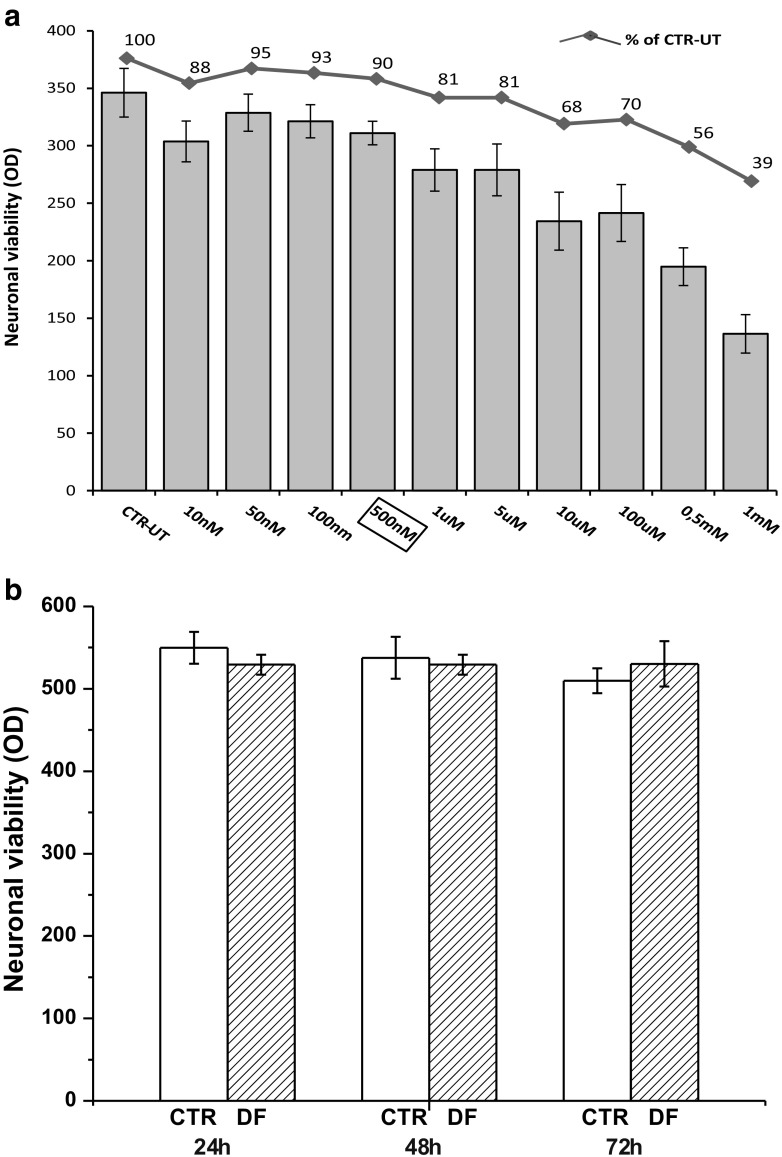
**a** Effect of different concentration of DF3016A on neuronal cortical cells. Values in the columns are means ± SEM of three different cultures. The line + symbol shows the same data as a percentage of vitality. **b** Viability of neurons treated with 500 nM of DF3016A for 24, 48, and 72 h

To evaluate the effects of DF3016A on neuronal viability we performed dose-response experiments with different DF3016A concentrations for 24 h. Data were obtained using the MTS assay from three different cultures; neurons exhibited dose-dependent changes in cellular viability (Fig. 1a). From 10 nM to 5 μM of DF3016A concentration, no significant mortality was observed compared to the control. On the basis of these results, the 500 nM concentration was chosen for performing the subsequent experiments, and cortical neurons were treated for 24, 48, and 72 h without significant viability changes (Fig. 1b).

### Cellular Viability After OGD-R Injury

**Fig. 2:**
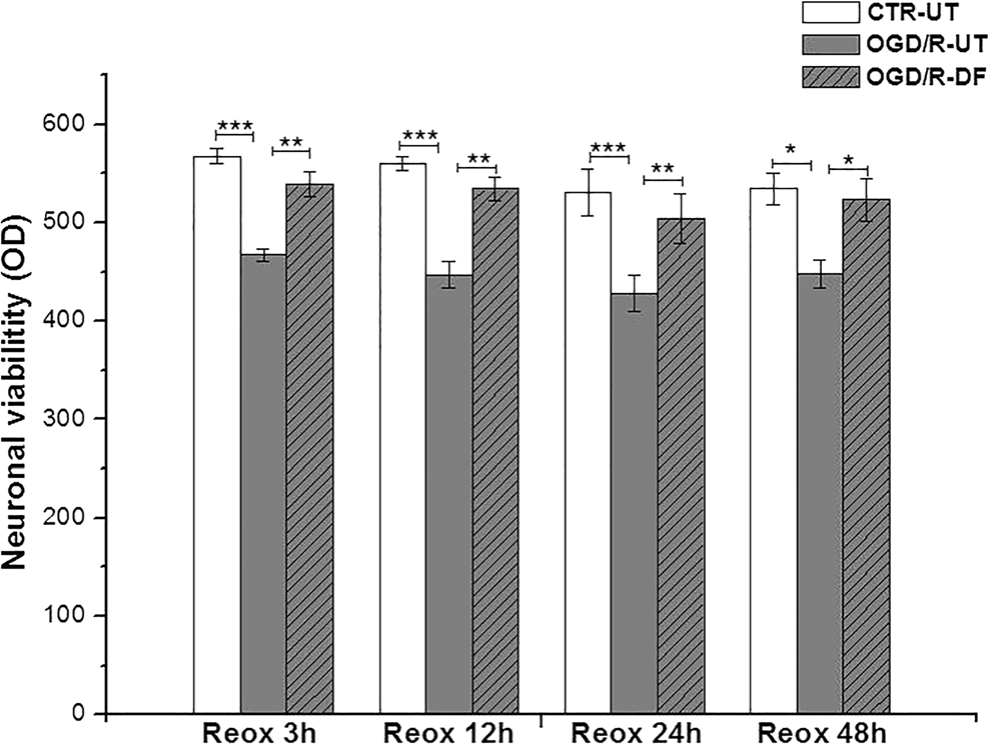
Cortical neurons treated with DF3016A 500 nM and exposed to OGD and different reoxygenation time points (3, 12, 24, 48 h). Data are means ± SEM of three different cultures. *P < 0.05, **P < 0.01, ***P < 0.001 (Bonferroni’s post hoc test). CTR-UT control-untreated, OGD/R-UT neurons exposed to OGD/R, CTR-DF control treated with 500 nM DF3016A, OGD/R-DF neurons exposed to OGD/R and treated with 500 nM DF3016A

**Fig. 3:**
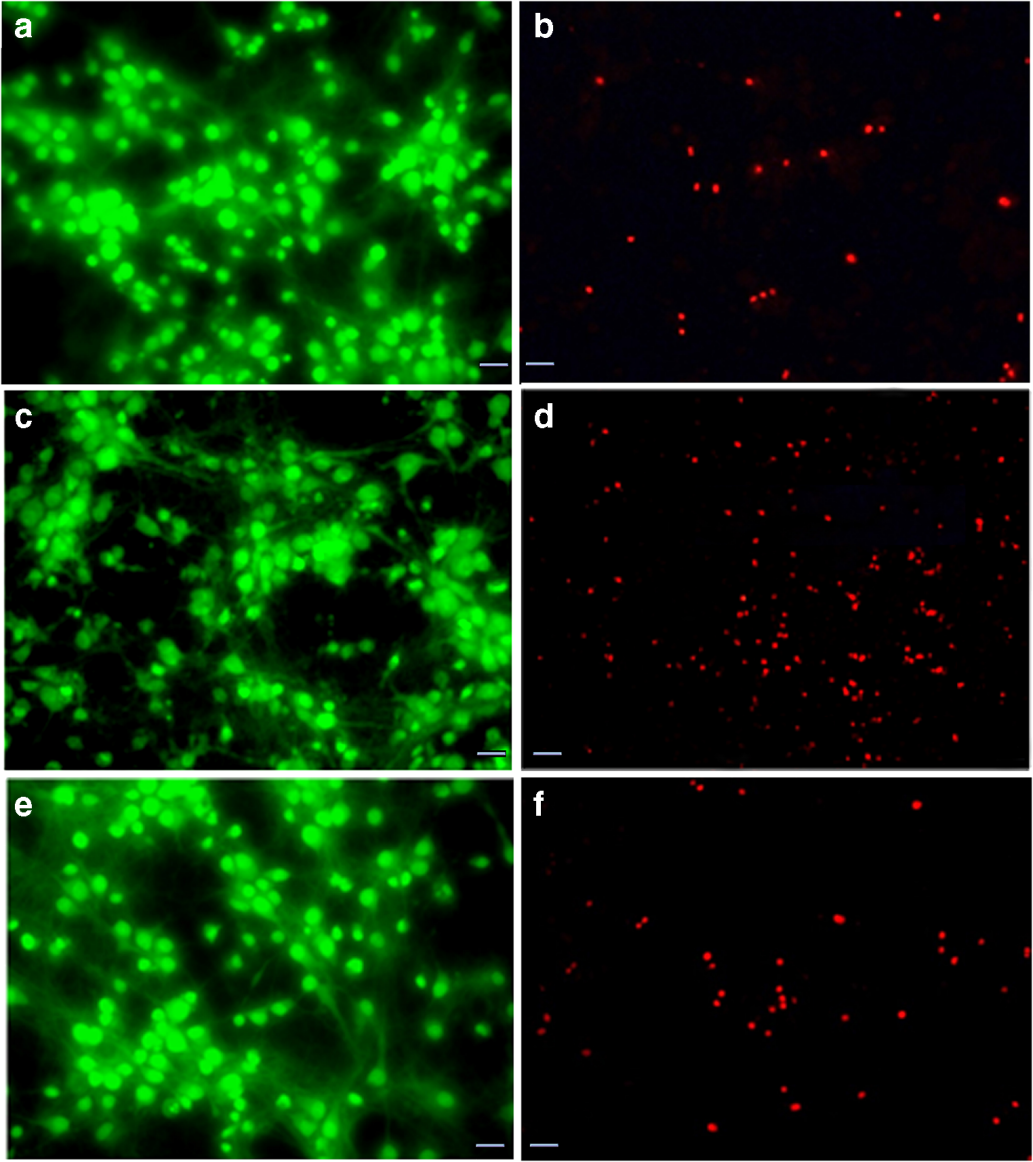
Acridine Orange/Ethidium Bromide (AO/ET) staining for discrimination of live and dead cells. **a**, **b** represent control neurons; **c**, **d** are neurons exposed to OGD/R 3 h; and **e**, **f** are neurons treated with 500 nM DF3016A and exposed to OGD/R 3 h

To examine the effect of DF3016A during OGD and at different time points of re-oxygenation, 500 nM of DF3016A was added to the culture medium. As estimated by the MTS assay, the viability of OGD/R cultures was reduced by about 20% compared to the control. Following 3 to 48 h of reperfusion, the treatment with 500 nM DF3016A significantly attenuated OGD/R-induced cell death (Fig. 2). As described, neuronal viability was also assessed using Acridine Orange/Ethidium Bromide (AO/ET) staining. Viable cells appear evenly green, in contrast to dead cells that appear as red spots due to chromatinic deposits. OGD/R exposure induced an increase in neuronal loss (Fig. 3c, d) while in ischemic neurons, treated with DF3016A, the neuronal death caused by OGD/R exposure was close to that of control (Fig. 3e, f).

### Effects of DF3016A Treatment on Neuronal Calcium Influx

**Fig. 4:**
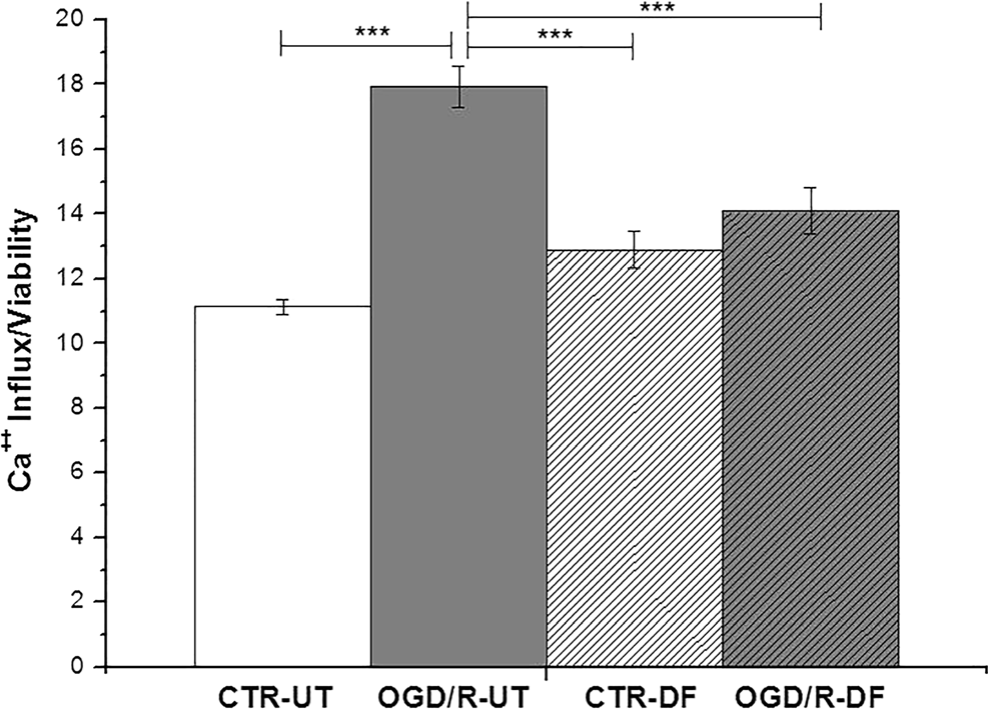
Calcium influx measured in control and DF-treated cultures exposed to OGD/R

C5aR has been suggested to also act as a modulator of Ca^2+^ influx via L-type voltage-gated Ca^2+^ channels (L-VGCCs) in neurons (Farkas et al. 2012). As shown in Fig. 4, OGD/R-UT exposure induced an abnormal Ca^2+^ influx (*P* < 0.001 vs non-exposed cells) related to neuronal loss (shown in Fig. 2) probably due to excitotoxicity events. Notably, the 500-nM C5a antagonist treatment triggered a significant reduction of Ca^2+^ influx in OGD/R neurons (*P* < 0.001 OGD/R-DF vs OGD/R-UT cells).

### DF3016A as an Antagonist on C5a And C5aR Expression

**Fig. 5:**
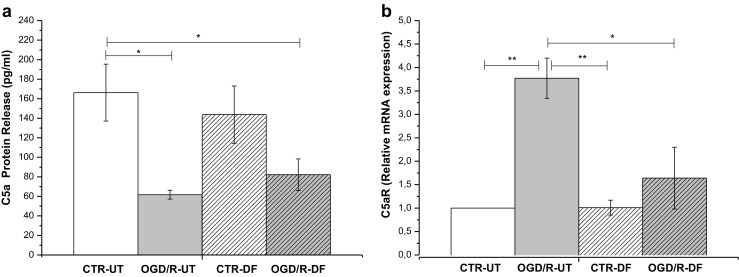
**a** OGD/R-UT condition dramatically reduced the C5a protein level but induced a significant increase of C5aR gene expression. **b** DF3016A C5aR antagonist notably reduced C5aR transcription (mean ± SEM six independent experiments). *P < 0.05, **P < 0.01, ***P < 0.000 (Bonferroni’s post hoc test). CTR/R-UT control-untreated, OGD/R-UT OGD/R exposed neurons, CTR/R-DF control treated with 500 nM DF3016A, OGD/R-DF OGD/R exposed neurons treated with 500 nM DF3016A

From the results obtained after OGD/R exposure, regarding the expression of C5aR and C5a protein release, we have observed a significant decrease of C5a released protein (Fig. 5a), tightly related to the C5aR over-expression (Fig. 5b). DF3016A treatment reverted C5aR gene expression to the control levels (Fig. 5b). Likewise, DF3016A restored C5a protein level to not significant values compared to control (Fig. 5a).

### DF3016A Effects on Pro-Inflammatory Cytokines

**Fig. 6:**
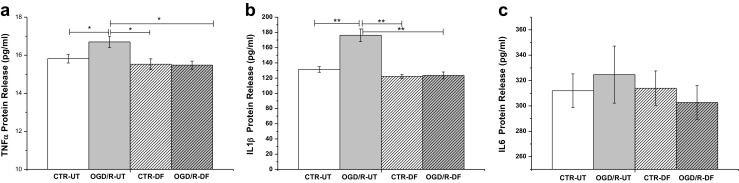
Graph showing pro-inflammatory cytokine release after OGD/R exposure and DF3016A treatment (mean ± SEM of four independent experiments; *P < 0.05, **P < 0.01) (Bonferroni’s post hoc test). CTR-UT control-untreated, OGD/R-UT OGD/R exposed neurons, CTR-DF control treated with 500 nM DF3016A, OGD/R-DF OGD/R exposed neurons treated with 500 nM DF3016A

Considering that C5aR activation largely contributes to inflammatory responses in several diseases leading to neurodegenerative conditions (Choudhry et al. 2016; Yanamadala and Friedlander 2010), we analyzed the major pro-inflammatory cytokine release after OGD/R exposure in neuronal cultures with and without 500-nM DF3016A treatment. ELISA analysis showed a significant upregulation of TNF-α (Fig. 6a) and IL-1β (Fig. 6b) during ischemic neuronal injury. The IL-6 release was higher in OGD/R, even if data were not significant (Fig. 6c). The effect on IL-6 was surprising. We presume that our sublethal stimulation was unable to elicit enough IL-6 expression in cortical neurons. Indeed, recent data demonstrated that high IL-6 levels are released in dorsal root ganglion cultures with high satellite glial cell levels (80%) and is stimulated by lipopolysaccharide release (Leisengang et al. 2018). C5aR antagonist treatment lead to a restoration of control conditions of ischemic/hypoxic neurons (Fig. 6a, b).

### Effects of DF3016A on miR-181a Expression

**Fig. 7:**
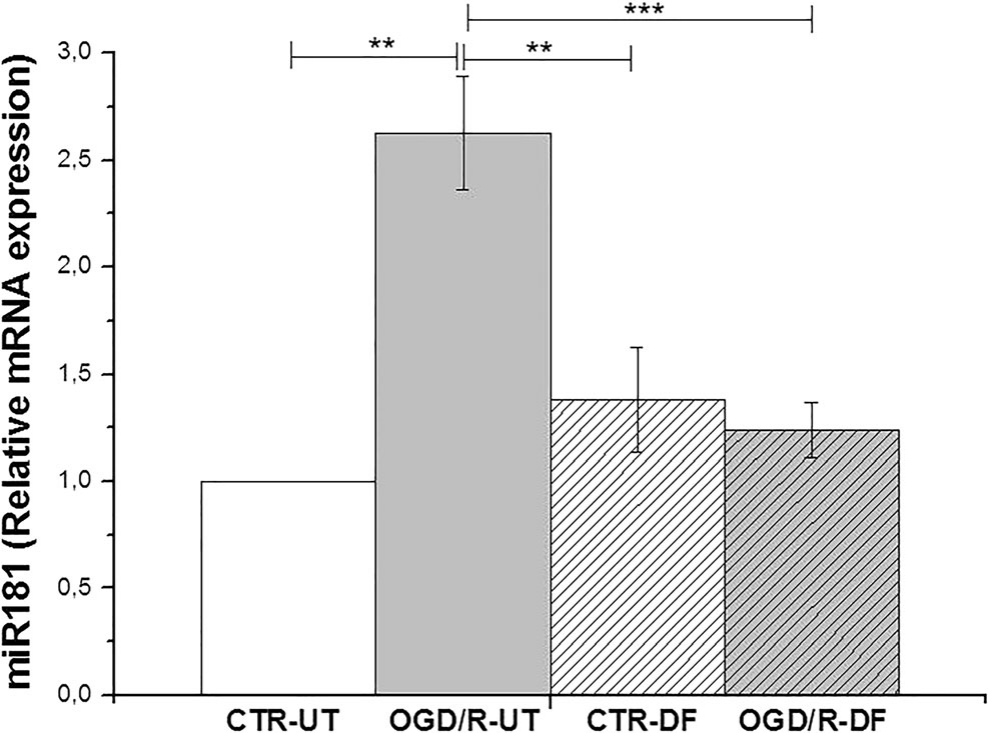
Graph showing the miR-181a relative gene expression levels under OGD/R condition and DF3016A treatment. Data from four independent experiments (mean ± SEM). **P < 0.01, ***P < 0.000 (Bonferroni’s post hoc test). CTR-UT control-untreated, OGD/R-UT OGD/R exposed neurons, CTR-DF control treated with 500 nM DF3016A, OGD/R-DF OGD/R exposed neurons treated with 500 nM DF3016A

MicroRNAs, small non-coding RNA molecules, play an essential role in the regulation of gene expression through the degradation/inhibition of mRNA targets. In particular, miR-181a seems to be actively involved in the response to inflammatory stimuli (Xie et al. 2013). OGD/R induced a significant upregulation of miR-181 expression (*P* < 0.01 vs non-exposed cells), but DF3016A treatment completely attenuated the miR-181a over-expression (*P* < 0.001 OGD/R-DF vs OGD/R-UT) (Fig. 7).

## Discussion

Neuroinflammation can involve activation of the brain immune system in response to an inflammatory challenge (Alexander et al. 2008). It is characterized by cellular and molecular changes within the brain that involve many immunological mediators such as the complement system (Van Beek et al. 2003). Neuroinflammation and the subsequent neurodegenerative processes have clear clinical consequences (Paladini et al. 2015; Paladini et al. 2016; Fusco et al. 2017; Zis et al. 2017; Zis et al. 2017a; Varrassi et al. 2018) Uncontrolled complement activation can lead to excess tissue inflammation and damage; moreover, neuroinflammation induces sensitization and synaptic hyperactivity in the peripheral and central nervous system (Varrassi et al. 2015).

Here we evaluated the effects of DF3016A, a new molecule that acts as a selective C5aR antagonist. C5a is a crucial terminal component of the complement cascade supporting nociceptive sensitization and inflammation. The results reported here show that DF3016A is a potent and specific inhibitor of C5aR (IC50 in the nanomolar range) with a high selectivity based on a panel of GPCRs. Furthermore, DF3016A is characterized as an orally bioavailable C5aR antagonist (rat bioavailability = 69%, oral *t*_1/2_ = 6.1 h) with good brain penetration (Kpuu = 0.44) upon oral administration. Based on this evidence, we further characterized the effects of the compound on primary rat cortical neurons exposed to sublethal OGD/R, an in vitro model of neuroinflammation-related processes. Firstly, we showed that treatment with DF3016A for 24 h in the concentration range 10 nM–5 μM did not affect neuronal viability and the toxic concentration of DF3016A (TC50 = dose that kills 50% of the cells) was between 0.5 and 1 mM. Additionally, a longer treatment for up to 72 h with 500 nM DF3016A did not affect significantly neuronal viability. We then tested the effect of the drug using the OGD/R model that produces deficits in synaptic function, which has been widely used as an in vitro model to mimic the effects of ischemia-reperfusion injury and neuroinflammation in neurodegenerative diseases. Our experimental protocol was based on the exposure of primary neuronal cultures to 1 h of OGD and 3 h of reoxygenation. The protocol also allowed us to study the early neuronal response, based on the finding of no significant differences in the viability of cultures kept in the reoxygenation state for up to 48 h. This insult induces a mild degree of neuronal death and allows investigation of the functional activity of viable neurons. During ischemia, an excess of calcium influx is believed to be one of the first events triggering excitotoxic cell death (Mukherjee and Pasinetti 2001; Farkas et al. 1998). The increase in intracellular calcium level is an important element in the signal-transduction network activated after the binding of C5a fragment complement to cell-surface receptors (Monk and Partridge 1993; Triantafilou et al. 2013). We observed that DF3016A treatment nullified the large increase of Ca^2+^ influx induced by OGD/R and restored calcium concentrations to control levels. Previous work has demonstrated that the increased levels of released C5a in neuronal cultures exposed to OGD are tightly linked to apoptosis (Pavlovski et al. 2012). In the present study, we did not observe any increase in release of C5a protein in OGD/R neurons. On the contrary, C5aR gene expression was upregulated after OGD/R treatment. This upregulation is an interesting and may suggest that our OGD/R model induces an early upregulation of C5aR expression, and subsequent binding of C5a to its receptor, explaining the lower levels of C5a protein observed. It is noteworthy that the complement pathway can be activated immediately after neuronal injury, and the increased receptor expression is one of the first steps in this response (Woodruff et al. 2010). Moreover, our results may not be in contrast to those previously observed after 12 h of OGD (Pavlovski et al. 2012), as the increase in C5a protein could be a subsequent process activated by more detrimental injuries, leading neuronal cells to apoptosis. Indeed, production of a proteolytic fragment of C5a is a late event in the complement cascade, regulated by many complement-regulatory molecules which are subject to control by the expression of several receptors such as inflammatory mediators (Woodruff et al. 2010; Van Beek et al. 2000). However, DF3016A treatment restores the control situation, with the expression of C5a maintaining the unchanged levels of the protein in OGD/R-treated neurons. As shown by different authors (Sayah et al. 1999; Song et al. 2018), C5a protein binding to its receptor can lead to the production of pro-inflammatory cytokines such as IL-1β, TNF-α, and IL-6 which in turn affect complement expression (Busch et al. 2013). In keeping with these pieces of evidence, in our experimental model, we observed overexpression of the major pro-inflammatory cytokines in OGD/R-exposed cultures. Remarkably, DF3016A treatment abolished the increase. The complex cytokine network in CNS is involved not only in the immune response but also in a variety of physiological and pathological processes (Szelényi 2001). Nevertheless, even though there are reports that low concentrations of cytokines can exhibit beneficial effects on neuronal viability and neurological function (Di Loreto et al. 2000; Vezzani and Viviani 2015), an injury-induced overexpression promotes neuronal death.

Increasing evidence supports a significant role for miRNAs in response to cerebral ischemia (Ouyang and Giffard 2013; Dharap et al. 2009). MiRNAs are small non-coding RNA molecules that control gene expression at the post-transcriptional level, and are abundantly expressed during brain development and in the adult mammalian brain (Kos et al. 2016). MiRNAs target messenger RNAs (mRNAs). MiRNAs can induce mRNA degradation or the repression of the process of translation to modulate gene expression, and a single miRNA can bind and regulate several mRNA targets. The miR-181 family, especially miR-181a and miR-181b, are enriched in the brain (Miska et al. 2004), and their aberrant expression has been associated with brain diseases. The knockdown of miR-181a enhanced the production of pro-inflammatory cytokines (TNF-α, IL-6, IL-1β, IL-8) (Hutchison et al. 2013), and increased levels of the anti-inflammatory cytokine IL-10 result from miR-181 over-expression (Murray 2005; de Vries 1995) in a murine stroke model. In a model of cerebral ischemia, the expression of miR-181 increases in the area where cell death occurs, whereas expression is lower in the penumbra zone where cells can survive (Ouyang et al. 2012). Altogether, these data indicate that miR-181 is involved in general transcriptional profiles of cellular stress response and cellular survival-related changes. Therefore, we focused on the investigation of miR-181a in our model. Our data revealed increases in miR-181a expression after OGD/R exposure that could represent an early neuronal response to ischemic injury. DF3016A treatment completely abolished this over-expression, suggesting that this C5aR antagonist could affect immune-mediator expression also through interactions with transcriptional factors such as miR-181a. The overall profile of DF3016A made it an excellent candidate for additional in vitro and in vivo studies to assess the pharmacological effects of a C5aR antagonist with good brain penetration.

## Conclusion

Altogether, these results support for the first time the hypothesis that DF3016A, a potent and selective C5aR inhibitor, might exhibit neuroprotective effects against neuroinflammatory processes induced by OGD/R in primary cortical neurons. This could have important clinical consequences. Our experimental model allowed the investigation of CNS molecular and biological mechanisms in a controlled environment in vitro and on individual cell types. We did not study potential interactions with different cellular phenotypes in the brain. In conclusion, this study suggests that DF3016A might be a candidate molecule for a novel therapeutic approach to neuroinflammation-related diseases. Further researches are in progress to evaluate the possible cellular and molecular mechanisms underlying the observed neuroprotection.
